# Development of a data-driven urban immunity assessment model: providing a new benchmark for urban governance under public health emergencies

**DOI:** 10.3389/fpubh.2025.1609641

**Published:** 2025-05-29

**Authors:** Peng Cui, Saiya Cao, Ruize Qin, Fan Zhang

**Affiliations:** ^1^School of Civil Engineering, Nanjing Forestry University, Nanjing, China; ^2^Department of Applied Physics and Electronics, Umea University, Umea, Sweden; ^3^Department of Industrial Engineering and Operations Research, Columbia University, New York, NY, United States; ^4^Department of Building and Real Estate, Hong Kong Polytechnic University, Kowloon, Hong Kong SAR, China

**Keywords:** public health emergencies management, urban immunity, machine learning, assessment system, optimization model

## Abstract

Public health emergencies (PHEs) pose significant challenges to global urban governance systems, necessitating the establishment of more efficient and dynamically adaptive response mechanisms. Numerous cases indicate that current urban governance still faces the risk of systemic failure under PHE shocks, leading to severe socio-economic consequences. Existing studies, based on theories such as resilience, emergency management, and risk management, primarily employ traditional statistical modeling or single-discipline approaches to explore improvement pathways. However, they fall short in cross-system and multi-agent coordination mechanisms, as well as data-driven intelligent optimization. Therefore, this project draws inspiration from the principles of the human immune system, introduces the concept of urban immunity to characterize the level of urban governance under PHEs, and follows the approach of “feature decoding → mechanism analysis → spatiotemporal measurement → trend prediction → model optimization → decision output.” It refines the theoretical framework of urban immunity, analyzes urban immune response mechanisms, develops an immunity indicator system, assesses the spatiotemporal patterns of urban immunity, and builds a decision-making model using intelligent optimization methods to generate optimized solutions for different scenarios. Ultimately, the project aims to establish a data-driven, evidence-based decision-making approach. This project seeks to provide a more systematic and operational theoretical framework for urban public health governance while promoting the digital and intelligent transformation of public health management, thereby enhancing PHE prevention and control capabilities.

## 1 Introduction

Although human beings have entered the “Post-Epidemic Era,” public health emergencies (PHEs) continue to affect urban governance and global public health security by virtue of their strong uncertainty, infectivity, concealment and urgency, and have a great negative impact on life, health and economic society (1). On average, the World Health Organization (WHO) reports more than 200 PHEs globally each year, and 377 in 2023, with more than 70% of them occurring in urban areas, including monkeypox, Ebola, avian influenza, Zika virus, influenza A, mass poisoning, and outbreaks of environmental pollution. The annual global economic loss due to infectious disease outbreaks amounts to $600 billion, accounting for about 0.7% of the global economy. COVID-19 has spread to 213 countries and regions since its outbreak in 2020, with more than 700 million cumulative confirmed cases and more than 7 million deaths, leading to a global economic loss of $22 trillion, which accounts for more than 20% of the global GDP.

To this end, governments and organizations around the world have formulated framework documents and policies to enhance the governance capacity of cities in responding to PHEs, such as: WHO issued the “Strengthening health emergency preparedness in cities and urban settings” to build a framework and strategy for urban health event preparedness, the United States introduced the “Global Health Security Strategy” to promote the construction of national health security systems and surveillance capacity, and Singapore has launched the “Smart Nation” plan to use sensors and data analytics to monitor the spread of infectious diseases and optimize resource allocation. China released “Sustainable cities and communities—Guidelines for public-health emergency response in smart city operating models,” aiming to realize efficient management of PHEs through smart city construction (2).

Meanwhile, scholars from various countries have proposed or introduced urban governance-related theories across fields, such as resilience theory, complex systems theory, multicenter synergistic governance theory, adaptive governance theory, etc., which provide unique research perspectives and broad-spectrum solutions for research on urban disaster governance and emergency management. These theories have been widely applied to different types of disaster management such as natural disasters, industrial accidents, and public safety crises. However, due to the unique triggering and propagation mechanisms of PHEs, there is a risk of governance failure in the responses and programs supported by traditional theories when cities face the impact of PHEs. For example, in 2012, the outbreak of fungal meningitis was caused by the contamination of injections produced by the New England Drug Company in the United States due to non-compliance with hygiene standards in the production process, loopholes in the regulatory system and the lack of a rapid recall mechanism; and in the 2014 Ebola epidemic in West Africa, poor public health infrastructure, slow government response, and the collapse of public trust led to the Liberian capital Monrovia ‘s failed public health governance system; at the early stage of COVID-19 in 2020, the healthcare system in Lombardy region of Italy collapsed due to a combination of imbalanced healthcare resource deployment, poor information communication, and ineffective synergy between the local and central governments; and the outbreak of dengue fever in China’s Guangxi’s Teng County in the second half of 2023 due to ineffective vector control, a shortage of primary healthcare resources, and a lag in the public disclosure of information.

Urban governance under PHEs is a complex and systematic project, and its operation mechanism is highly similar to the process of the human immune system against pathogens. Each functional department plays a role in the governance system similar to that of different types of immune cells in the immune system, and through efficient synergy and precise linkage, they work together to construct the first line of defense for urban public safety. The information transmission between different levels of government can be likened to the signaling of cytokines in the immune system. When external threats appear, the governing body needs to respond quickly, identify the risks accurately, and efficiently integrate and dispatch resources to ensure the timely implementation of preventive and control measures. The material deployment mechanism is similar to the rapid aggregation and targeted response of antibodies and immune cells, aiming at guaranteeing the normal operation of key defense and control departments and enhancing the overall anti-risk capacity of the city. Through this dynamic feedback and resource optimization mechanism, the urban governance system continuously strengthens its adaptive capacity in the synergistic interaction of multiple subjects and levels, and ultimately achieves effective defense and rapid recovery from PHEs.

From this association, can we draw on the operating principle of the human immune system to put forward the brand-new concept of “urban immunity” and apply it to the urban governance under PHEs? Compared with traditional concepts or frameworks, the concept of urban immunity and its corresponding system have the following advantages: (1) a clearer theoretical source, greater situational applicability and enhanced relevance, with the three phases of “defense, response, and recovery” at its core, emphasizing dynamic changes and differentiated strategies at different stages; (2) better systematicity and coordination, similar to the synergistic operation of human immune cells, emphasizing the coordination ability among various systems (medical care, emergency management, community organizations), which is especially suitable for multi-sectoral cooperation in public health governance; (3) a more reasonable system of assessment indexes, which characterizes the level of urban public health governance in terms of immunity, and allows for the design of quantitative indexes that are more detailed, clearer, and more grounded; and (4) creatively introducing the “immune memory mechanism” to improve the ability of cities to cope with PHEs in the future through experience accumulation of historical events (such as policy optimization and technology upgrading).

Therefore, this study introduces the concept of “urban immunity,” inspired by the principles of the human immune system’s response to pathogens, offering a novel theoretical framework for urban governance. It effectively complements and extends traditional theories of resilience governance and emergency management. The research integrates system dynamics (SD), agent-based modeling (ABM), knowledge graphs, graph neural networks (GNN), graph convolutional networks (GCN), and deep q-network (DQN) to construct a unified framework for comprehensive simulation, evaluation, and optimization of urban emergency response. Furthermore, the urban immunization optimization model (UIOM) developed in this study is capable of generating specific, actionable strategies for various public health emergency scenarios, while incorporating social equity indicators to support more inclusive and scientifically robust policy formulation.

## 2 Literature review

The study of urban immunity under PHEs is an intersection and extension of theories and methods in multidisciplinary fields such as risk management, emergency management, urban governance, public health governance, and immunology principles, focusing on both the effectiveness of emergency management and the improvement of multisectoral collaboration capacity of cities in crises, as well as the urban practice of public health governance strategies such as public health resource scheduling, and risk blocking, and also how to bring immunity as an interdisciplinary concept into the field of management.

Resilient governance theory, as a mainstream theory of urban governance, systematically focuses on how to rapidly recover and adapt under external shocks or pressures, emphasizing multi-level coordination of the governance system, self-adaptive capacity, and long-term planning in the face of future crises (3, 4). The core of adaptive governance theory lies in the support of continuous learning, adjustment of strategies and cooperation among governance subjects in dynamic changes when dealing with complexity and uncertainty, which emphasizes cross-level and cross-subject cooperation, real-time feedback mechanisms and self-adjustment capabilities to ensure continuous optimization of governance in changing environments, and focuses on the use of socio-ecological systems to respond to changes in environments and to flexibly adjust governance strategies, which is suitable for coping with Complex and unpredictable public problems (5). Complex systems/network governance theory focuses on nonlinear behaviors, self-organization, dynamic changes, and interdependence among systems in a system, and achieves governance goals through networked cooperation (6–7). Collaborative governance theory investigates how to effectively govern and coordinate among multiple interacting actors to better address unexpected social challenges through the advantages of “weak centrality” such as multi-actor participation, decentralized decision-making, information flow and transparency, flexibility and adaptability (8). Participatory governance theory focuses on encouraging all members of society, especially groups that are typically excluded from decision-making, to participate in the governance process, emphasizing broad popular participation to promote more inclusive and equitable social outcomes (9). Polycentric governance theory, on the other hand, focuses on the dispersion of power and interactions between multiple governance levels and subjects, emphasizing that governance should not be dominated by a single central government, but rather through multiple self-organizing, interconnected governance units (10). Smart governance relies more on technology and digital platforms, focusing on the use of modern technology to enhance the ability of the government and all social parties to deal with complex social problems, combining technologies such as information technology, artificial intelligence, and the Internet of Things to promote the intelligence, synergy, and sustainability of public governance (11). Relying on the above theories, a series of emergency response frameworks have been formed internationally, the core of which lies in optimizing the governance structure so as to achieve multi-level collaboration, information sharing and systematic dynamic response, and ultimately enhance the overall emergency management capacity (12). The experience during COVID-19 has shown that outbreak mitigation policies (e.g., maintaining social distance) play a key role in reducing the risk of early disease transmission, informing future public health event responses (13). For example, the Governance Framework for Public Health Pandemics (GGPC) further refines the governance process, covering six phases: risk identification, early warning response, emergency response, recovery and reconstruction, long-term adaptation, and targeted governance, providing systematic governance solutions for different countries and regions (14). In addition, the United States, Japan, and Germany have established a unified command structure in their emergency management systems, which enables the government and various social actors to collaborate in responding to outbreak challenges (15).

The level of urban governance under PHEs is influenced by the dynamic interactions of various factors such as infrastructure, economic systems, healthcare and emergency supplies distribution, and social networks (16, 17), while the performance of these factors in the city’s response to PHEs also determines the level of urban governance. For example, the load level of healthcare infrastructure during epidemics affects various indicators such as healthcare resource allocation, emergency preparedness, and treatment efficiency (18); the surge in hospital loads leads to capacity diversion and reduced efficiency of municipal functions such as public transportation and community services (19); and the blockade policy and stagnation of economic activities lead to a dramatic changes in the structure of energy demand, which has led to a significant decline in commercial and industrial electricity demand and a significant rise in household electricity demand (20, 21). With transportation constraints and delays in energy and material supply chains, energy supply–demand imbalances can trigger logistical disruptions and limited supply chain functioning, which puts even more pressure on city operations (22). Under PHEs, policy response becomes an important regulatory tool for the recovery of the economic system. For example, in the early stage of the epidemic, China adopted a strict embargo policy and effectively prevented the systemic collapse of the socioeconomic system through economic interventions such as industrial support and financial subsidies (6). Strengthening cross-sectoral collaboration mechanisms and integrating the resources of the government, healthcare institutions and community organizations to achieve rapid and efficient emergency response and dynamic dispatch (23). Enhance grassroots emergency response capacity through efficient emergency management mechanisms, including emergency prevention, rescue, decision-making, command, learning and accountability (24).

The empowerment of digital technology and the application of information technology has become an important transformational direction for urban governance, focusing on key areas such as policy formulation, drug development, and dynamic tracking (25). It also provides key support in terms of assessing and even predicting the risk of catastrophic events (26), which enhances the ability of cities to regain equilibrium after crisis disruptions by accurately identifying threats and optimizing resource allocation (27). For example, digital technological indicators (e.g., travel cards, health codes, and accurate community categorization) play an important role in pandemic prevention and control, providing technical support for accurate tracking and risk assessment (28, 29). It is widely used in the field of epidemic risk assessment in conjunction with machine learning and deep learning methods, and its efficient prediction capability and explanatory analysis help provide a scientific basis for emergency decision-making (30). By simulating the interactions among individuals, organizations, and environments, multi-intelligence body simulation dynamically analyzes the implementation effects of different governance strategies and evaluates the impact of these strategies on resource allocation and policy implementation efficiency (8). In addition, database system theory provides stable support for big data management and improves data storage and query efficiency (31). In terms of emergency decision optimization, Bayesian network-based scenario derivation methods have performed well in dealing with emergency strategy formulation under uncertainty and limited resources (32). Real-time data collection and sharing techniques have drastically reduced the early warning and response time of emergencies and improved governance efficiency (33). Geographic information system (GIS), as an important tool for epidemiological analysis, can be used to analyze epidemics from multiple dimensions, such as time, population, space, region, pathogen category, and quality of case reports, and provide a scientific basis for making precise defense decisions (34). With the arrival of the 5G era, the wide application of information and communication technologies will further enhance the resource scheduling efficiency and public health emergency decision-making capability, providing stronger technical support for the development of digital governance system (35).

“Immunity” refers to the physiological function of a living organism to maintain homeostasis through the recognition of self and foreign antigens by the immune system and a series of immune response mechanisms to remove potential threats. As the concept of immunity gradually expands to the fields of social immunity, economic immunity and organizational immunity, the field of management science also tries to find new research perspectives for risk management and emergency response through this concept. The managerial application of the immunity principle is based on the high similarity between the biological immunity mechanism and the organizational management system, which can effectively guide organizations on how to cope with external threats and maintain the normal operation of internal functions, as well as help them to classify and judge risks so as to select appropriate countermeasures through the crisis management, risk assessment, and early warning mechanisms (36). In recent years, the concept of immunity has been gradually demonstrated in the study of cities’ response to external crises, emphasizing rapid response, social stability, resource deployment, and post-disaster recovery, and the formation of an “immune response network” through policy iteration and resource integration to enhance the city’s defense and response capabilities against emergencies and cyberattacks (37), ensuring the resilience and sustainability of cities in complex systems (38, 39). Meanwhile, research perspectives and strategies such as the construction of urban herd immunity, identification of and response to immune blindness, considering social distancing and other preventive measures as “urban antibodies,” and enhancing artificial immunity through exercises further enhance the resilience of cities in complex environments (40, 41).

In summary, the current research related to urban governance under PHEs and the cross-disciplinary migration of the concept of immunity provides an important theoretical basis and practical guidance for exploring urban immunity, which is mainly reflected in the following aspects:

(1) Multidisciplinary cross-fertilization provides the research basis for this study. Urban immunity research under PHEs has gradually shown a trend of multidisciplinary cross-fertilization, covering a variety of fields such as risk management, emergency management, urban governance and public health governance. This interdisciplinary convergence has provided a theoretical basis for understanding the adaptability, resilience and emergency response of cities in public health crises, as well as promoted the application of related technologies in governance practices. This study will integrate and systematically model existing theories on the basis of interdisciplinary convergence to explore a more systematic and synergistic urban immunity governance framework.
(2) Multidimensional development of urban governance theory provides the theoretical foundation for this study. Urban governance theory has gradually transformed from the traditional linear management mode to the complex system management mode. Resilient governance emphasizes the adaptability and resilience of the system, evidence-based governance focuses on data-driven scientific decision-making, and collaborative governance emphasizes the joint participation of multiple subjects. Different theoretical frameworks have different application scenarios in urban governance, and the response to PHEs requires the integrated application of these theories to build a more dynamic, flexible and efficient governance system. This study will explore the applicability conditions of different governance modes and propose a more adaptable and dynamically adjustable theoretical system in combination with the real needs.
(3) The application of intelligent technology in urban governance provides a means of realization for this study. The application of big data, artificial intelligence, GIS and other intelligent technologies in urban governance and public health management has been deepening, improving the response speed and decision-making precision of emergency governance. These technologies play an important role in epidemic spread prediction, resource deployment optimization and risk assessment, making urban governance evolve gradually from traditional empirical decision-making to data-driven intelligent decision-making. This study will explore how to build a governance model of cross-technology integration and systematization with the support of intelligent technologies to improve the science and applicability of emergency governance.
(4) The interdisciplinary application of the concept of immunity provides an innovative perspective for this study. The concept of immunity has been extended from biology to management science, and has been applied to some extent in risk management, quality management, emergency management, etc. It provides an analogous analytical framework for understanding how complex systems resist external shocks and restore stability. However, current research focuses on theoretical discussions and lacks quantitative assessment methods and systematic analytical tools, resulting in the concept of immunity being applied in practice in a more abstract way and not yet resulting in actionable policy tools. Based on the logic of management application of immunity, this study will explore more specific governance mechanisms under this conceptual framework to promote the deepening and practical application of immunity theory in the field of urban governance.

Building upon this foundation, this study further refines the concept of “urban immunity,” constructs a theoretical framework, integrates a robust methodological system, develops assessment tools, and provides policy recommendations. These efforts aim to drive the transformation of urban governance from traditional emergency management to intelligent and resilient governance, offering a novel perspective on the sustainable development of cities and the safeguarding of public health.

## 3 Methods

**Figure 1:**
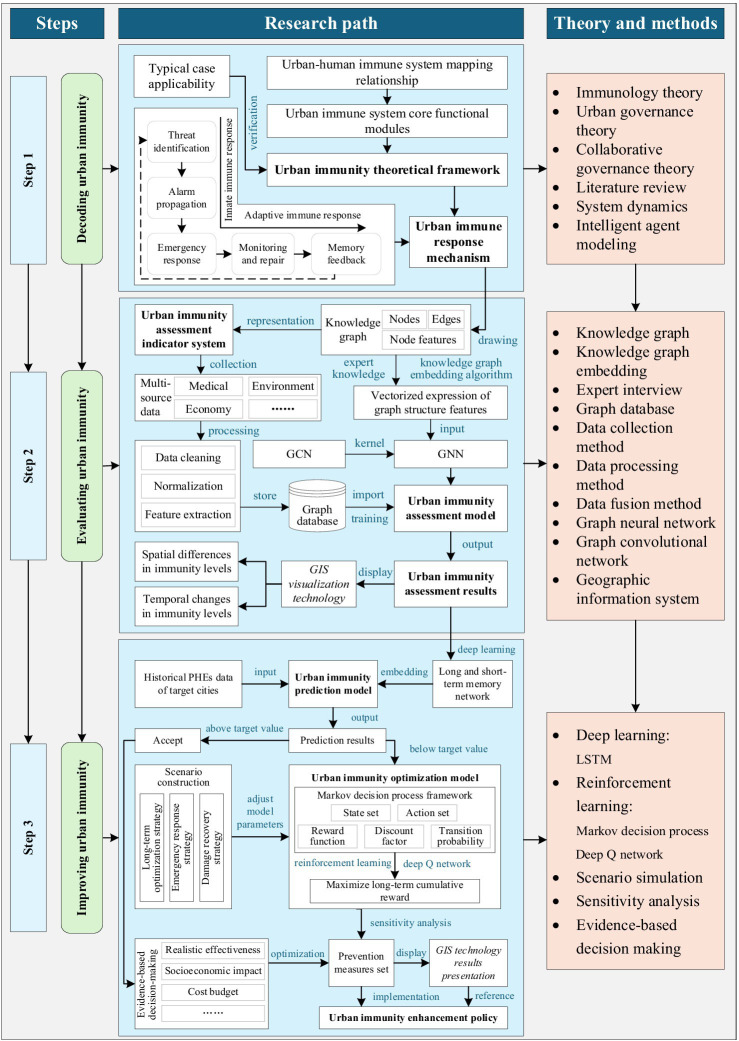
Technology roadmap.

From the perspective of the intersection of system science and public health governance, and with reference to the operation principle of the human immune system, this study innovatively puts forward the concept of “urban immunity,” which compares the response ability of cities in PHEs to the immune function of the human body, and constructs a dynamic conceptual framework that covers the threat identification, alert dissemination, emergency response, monitoring and repair, and memory feedback. At the same time, this study takes data-driven as the core, integrates multi-source data and artificial intelligence technology, systematically constructs a quantitative assessment and intelligent optimization system of urban immunity, and promotes the transformation of the governance scheme from empirical judgment to precise intervention; and promotes the transformation of the governance of PHEs from passive response to active adaptation and long-term optimization according to the three steps of “decode-evaluation-promotion,” as shown in Figure 1.

### 3.1 Step 1: decoding urban immunity

By comparing the human immune system with the urban governance system under PHEs, (1) we construct the theoretical framework of urban immunity, and (2) analyze the full-cycle response process of urban response to PHEs, so as to lay the theoretical foundation for the subsequent research on the assessment and enhancement of urban immunity.

#### 3.1.1 Theoretical framework of urban immunity

**Figure 2:**
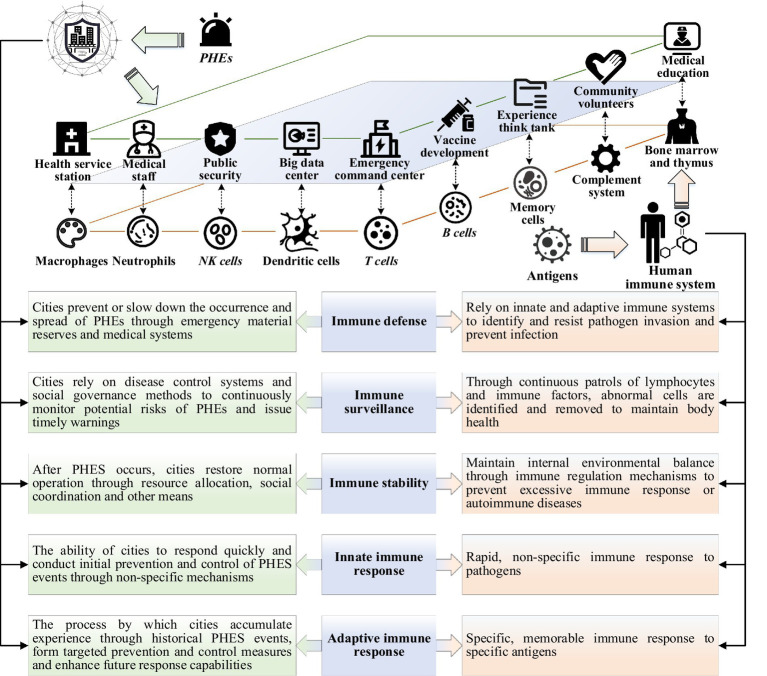
Conceptual mapping relationship between human and urban immunity.

First, according to the operation law of the human immune system and the urban public health governance process, the conceptual, connotative, functional, logical and other mapping relationships between the city and the human immune system are clarified, as shown in Figure 2. For example, the health service stations and big data center find epidemic analogous to macrophages and dendritic cells recognizing pathogens; the emergency command center coordinates multi-departmental responses, releases risk information, and activates the urban emergency response plan analogous to the immune system releasing inflammatory mediators, triggering local inflammation, and attracting immune cells to reach the site of infection; after the emergency is over, the city summarizes the emergency response experience and builds long-term governance capacity analogous to the human body initiating specific immunity and forming immune memory, etc. This mapping is not only a functional analogy, but also a systematic representation based on the interaction mechanisms between different entities (e.g., government, medical institutions, communities) and immune cells within the immune system. Through this mapping, the dynamic feedback mechanism of the immune system can be clearly explained in terms of its correspondence to coordinated responses, information transfer, and resource allocation in urban governance. Then, we define five core functional modules of the urban immune system, such as threat identification, alarm dissemination, emergency response, monitoring and repair, and memory feedback, and sort out the key elements such as the task behavior and information transmission path of the stakeholders of each module, so as to construct the theoretical framework of urban immunity. For example, in the threat identification module, hospitals adjust the detection rate and report case data in a timely manner after monitoring abnormal cases; in the emergency response phase, the government deploys resources and mobilizes supplies. In the model design, we ensured that the decision paths in each segment could be clearly explained, and provided explicit feedback mechanisms for decision-makers to help them understand how to respond based on different input factors. Finally, the historical cases of typical cities responding to PHEs are used as an entry point to check the cross-regional applicability of the urban immunity mapping relationship by combining the level of urban development, zoning and resource distribution, etc. The mapping relationship is validated and the theoretical framework is optimized for different urban characteristics to enhance its applicability in a variety of urban governance scenarios.

#### 3.1.2 Mechanisms of the urban immune response

**Figure 3:**
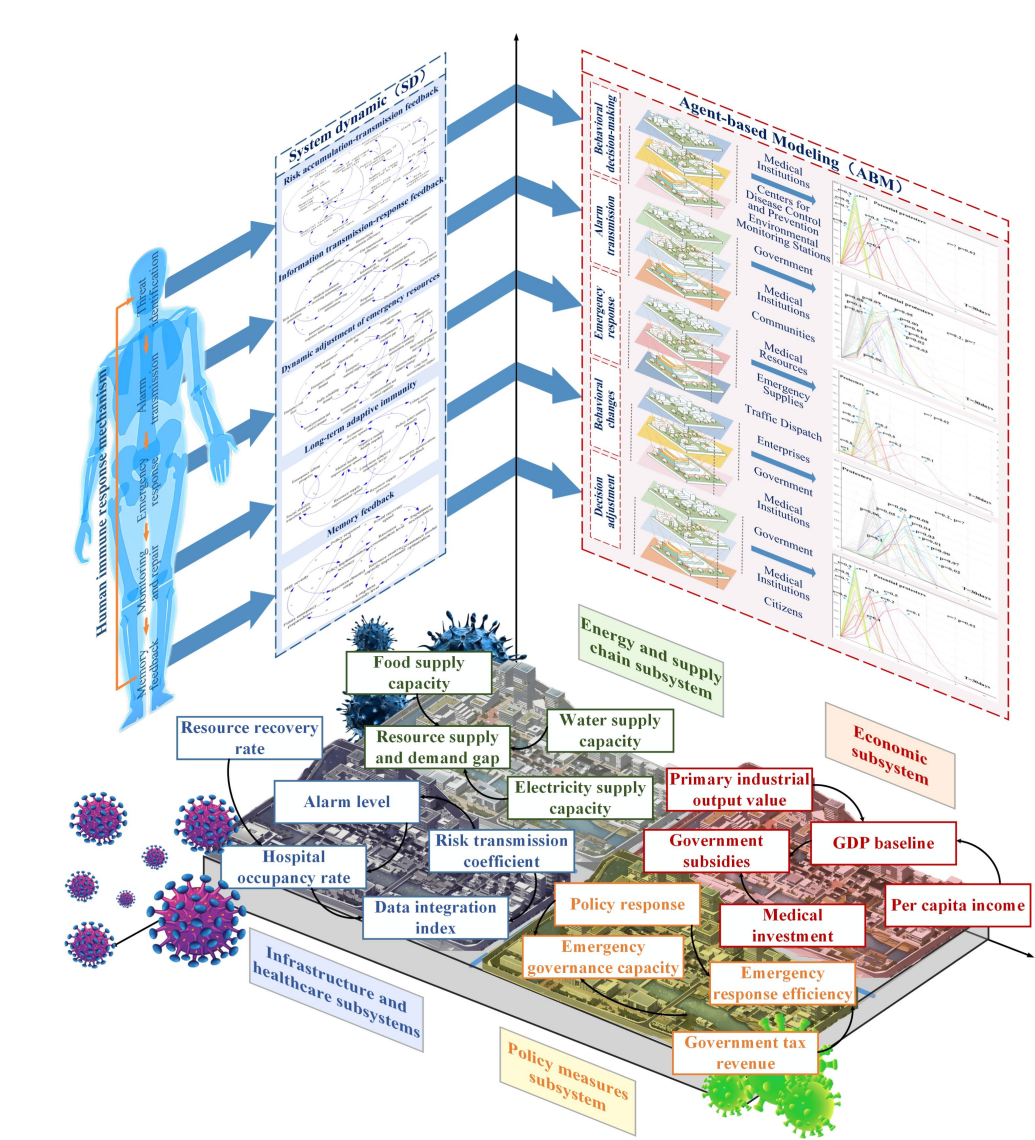
Mechanisms of the urban immune response.

The urban immune response can be divided into two stages: the intrinsic immune response and the adaptive immune response. The former is a rapid, basic, and non-specific defense mechanism spontaneously activated at the beginning of PHEs to achieve effective containment of early risks; the latter complements the intrinsic immune function through precise analysis, hierarchical management, resource integration, and the establishment of long-term memories to form specific, systematic, and long-term coping capacity. Based on the functional mapping relationship and theoretical framework of urban immunity, this part adopts SD to construct the feedback regulation mechanism of the urban immune system, and uses ABM to simulate the behavior and decision-making modes of different subjects, and the two are integrated to construct the urban immune response mechanism, and explore the interaction relationship between the functional modules at different stages, as shown in Figure 3. First, this study defines shared state variables (e.g., healthcare resources, infection rate) and synchronizes them between SD and ABM via a graph database, so that the intelligent body behavior influences the SD variable update, and SD feeds back the global state to constrain the intelligent body decision. Second, match the computational methods. The SD uses differential equations to simulate continuous variable changes, the ABM drives intelligent body behaviors based on rules or reinforcement learning, and sets a time-step coordination mechanism, e.g., the SD is updated on a daily basis, and the ABM simulates individual decisions on an hourly basis. This design ensures consistency between the two modeling approaches at both the time and decision levels, thereby enhancing the applicability and accuracy of the model in real-world scenarios. Finally, optimize the operational logic. Bidirectional interaction is realized through the trigger mechanism (e.g., ABM adjusts the policy when medical resources are below the threshold, and SD dynamically adjusts the infection rate after the upgrading of prevention and control measures), and during such interactions, the adjustments and outcomes of each decision are communicated to the decision-makers in a timely manner through a feedback mechanism, helping them understand the rationale behind the changes and ensuring the model’s adaptability and transparency. The details are as follows:

Threat identification: Its logic is analogous to the process of pathogen identification by the human immune system, aiming to analyze how cities can achieve early detection of risks such as epidemics, pollution, shortage of medical resources, through multi-source data monitoring (hospitals, centers for disease control and prevention, environmental monitoring stations, etc.), and improve the dynamic perception of potential threats. Among them, the SD model is used to establish the risk accumulation-propagation feedback mechanism, portray how risk factors spread in time and space, and express the coupling relationship between different monitoring points to optimize the risk identification accuracy; the ABM model is used to simulate the behavioral decision-making patterns of the government, hospitals, communities and other subjects in the process of risk perception, and to analyze the way different subjects interact with information in risk identification and their sensitivity to policy adjustments.

Alarm dissemination: Its logic is analogous to the inflammatory signal release and immune cell activation mechanism of the human immune system, aiming to analyze the transmission mechanism of risk information among multiple subjects such as the government, healthcare institutions, and the community, to explore the efficiency of information transmission, coordination of decision-making, and timeliness of response, and to simulate the transmission path of the alarms in the urban crisis management to ensure that the critical information reaches the decision-making level quickly. Among them, the SD model is used to construct the information dissemination-response feedback mechanism, analyze the timeliness of information flow in different transmission structures (e.g., hierarchical and flat), and the hierarchical response mode of the governmental decision-making chain; the ABM model is used to simulate how subjects such as the government, healthcare institutions, and communities receive, filter, and transmit the alert information, and to simulate the information turnover rate and information decay phenomenon under different governance structures.

Emergency response: Its logic is analogous to the human immune system’s process of clearing pathogens and regulating immune responses through immune cells and complement proteins, aiming to study the urban emergency response strategy in emergencies, optimize medical resource allocation, material dispatch, traffic control, and social prevention and control measures to ensure that the optimal strategy is adopted at different risk levels, while balancing the short-term prevention and control effects with the long-term socio-economic impacts. Among them, the SD model is used to establish a dynamic adjustment mechanism for the supply and demand of emergency resources, simulate the time dynamics of key elements such as medical resources, emergency materials, and traffic scheduling, and design the resource allocation strategy; the ABM model is used to simulate the behavioral rules of the government, hospitals, communities and other subjects in the emergency response, analyze how the resource allocation strategy affects the social stability, and assess the roles of different governance subjects in the preventive and control measures, and design a mechanism to match the supply and demand of resources.

Monitoring and restoration: This process is analogous to human cytokines regulating immune responses while activating the adaptive immune system, aiming at exploring the urban recovery process after emergencies, establishing the path of socio-economic restoration and how to gradually lift the state of emergency, restore the economy, optimize the mental health interventions to ensure a stable transition of the society, and form a long-term adaptive immune mechanism. Among them, the SD model is used to construct an adaptive immune feedback mechanism for the recovery process, simulate the city’s economic recovery speed, mental health indicators, resource supply pressure and other key factors in different recovery stages, and design the adjustment strategies in the restoration process; the ABM model is used to simulate the behavioral adjustment patterns of enterprises, governments, medical institutions, and citizens in the process of epidemic recovery, and to analyze the impact of key decision-making variables (e.g., financial subsidies, psychological intervention policies) on the speed of social recovery to ensure the efficiency and sustainability of the recovery mechanism.

Memory feedback: This process is analogous to the immune memory and dynamic adaptive capacity of the human immune system, aiming to study how cities summarize their experiences, optimize their governance model, enhance their resource reserve capacity, and establish a long-term immunity enhancement mechanism after experiencing PHEs, to ensure a faster response, more precise policy implementation, and stronger social adaptive capacity in future emergencies. Among them, the SD model is used to construct a long-term feedback mechanism of emergencies-policy response-social recovery-experience summarization, and analyze the government’s decision-making adjustment mode after multiple rounds of events; the ABM model is used to simulate the learning process of the government, healthcare institutions, and citizens in long-term immunity enhancement, and to analyze the changes in social acceptance of epidemic prevention policies and the effectiveness of long-term health governance strategies.

During the construction of the model, data from historical PHEs, such as emergency response data from influenza and SARS, are used to initially calibrate the model. Key parameters, including the infection rate, transmission rate, and resource consumption rate, are adjusted based on actual conditions. By comparing the model’s output with historical data, the model parameters are optimized to minimize the error between the model predictions and the actual emergency response data. To ensure the stability and reliability of the calibrated model, a cross-validation method is employed, where the dataset is divided into a training set and a test set to evaluate the model’s performance across different datasets and confirm its validity and stability under various scenarios.

### 3.2 Step 2: urban immunity assessment

(1) Draw the knowledge map as the indicator system for urban immunity assessment, (2) use deep learning technology to build a quantitative urban immunity assessment model, (3) Input the collected and preprocessed data into the model for training, and (4) Present and analyze the assessment results in time and space dimensions.

#### 3.2.1 Urban immunity assessment index system

Based on the elements of each functional module in the urban full-cycle immune response mechanism, the knowledge graph is used to construct the urban immunity assessment index system. Firstly, various types of intelligences, such as medical institutions, government departments, environmental monitoring systems, communities, are taken as the entity nodes of the knowledge graph, and the interactions of medical resource supply, epidemic spread, policy implementation, resource flow, and medical service distribution are taken as the relationship edges between the nodes, to form a graph network reflecting the interaction structure of the key functional modules of the urban immune system. In constructing the indicator system, particular attention must be given to the needs and equity of different social groups to ensure fairness in the distribution of resources and the implementation of policies, thereby preventing the neglect of vulnerable groups in the emergency response process. Secondly, semantic modeling is carried out by combining heterogeneous data from multiple sources, such as policy documents, medical data, epidemic reports, social media information, and unstructured information is transformed into a standardized knowledge graph structure through entity recognition and relationship extraction, and using graph databases to store and manage graph-structured data. On this basis, the Knowledge Graph Embedding (KGE) algorithm is used to vectorize the representation of entities and relationships, so that the immunity-related metrics (e.g., number of hospital beds, policy implementation rate, pollutant concentration) of each node can be efficiently quantified in the form of feature vectors, thus mapping the high-dimensional graph structure features of the urban immune system to a low-dimensional vector space. These embedded vectors will be used as high-quality inputs for downstream machine learning and deep learning tasks, effectively improving the accuracy and interpretability of urban immunity assessment and prediction, and supporting further structured analysis and decision optimization.

#### 3.2.2 Urban immunity assessment model

**Figure 4:**
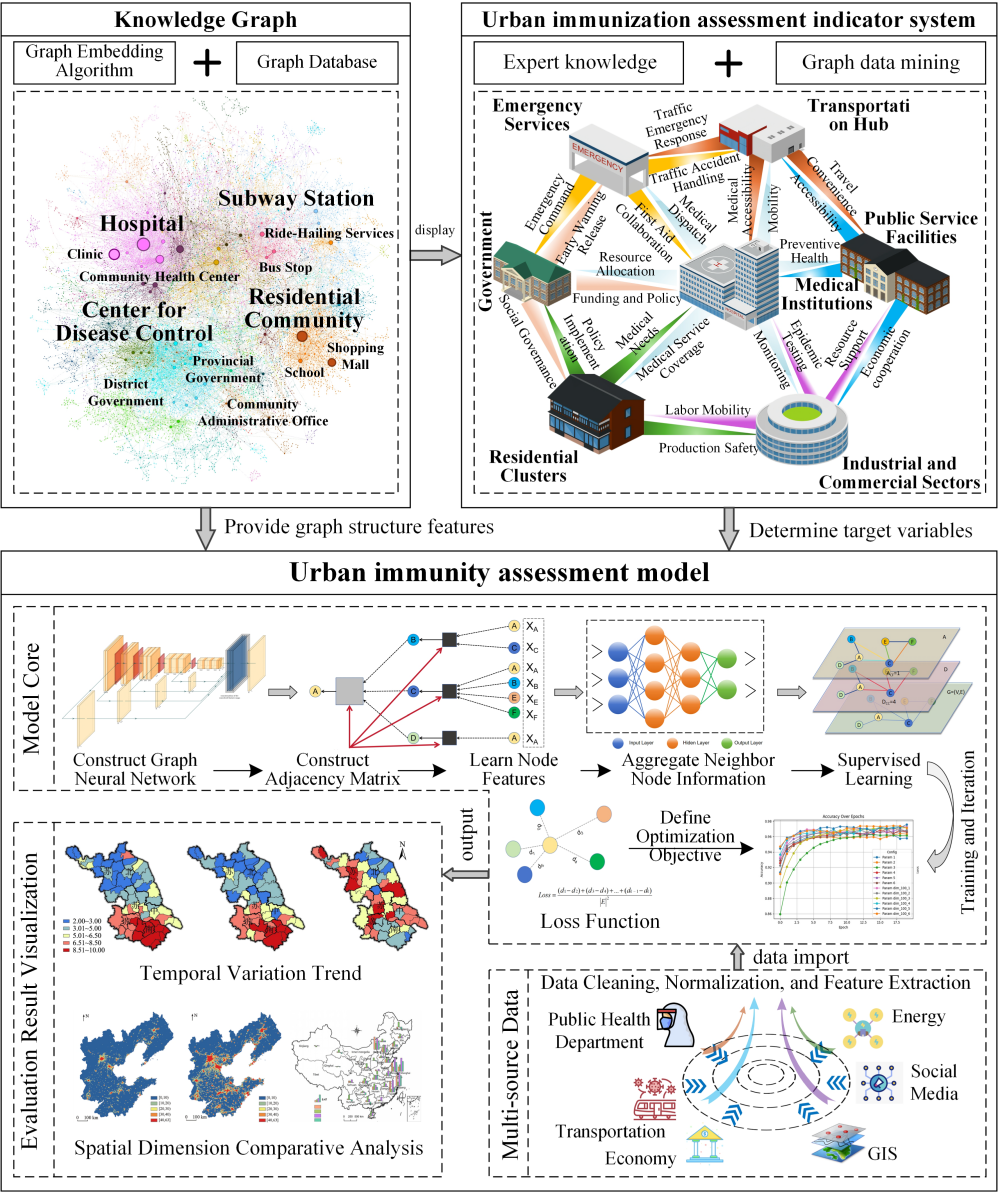
Urban immunity assessment framework.

Based on the knowledge graph, GNN are used for immunity assessment modeling, and GCN deep learning is selected as the core of the model, which is used to learn the correlation between urban immunity indicators and extract the intrinsic links between node features. Feature extraction and aggregation of graph data is performed by multilayer convolutional operations to predict the immunity level of the nodes and perform a comprehensive score as shown in Figure 4. First, aggregating neighbor node information and learning urban immunity features enable the model to predict the immunity scores of urban functional modules. Second, a neighbor matrix is constructed in the model training design to quantify the strength of ties between nodes, and a normalization matrix is used for preprocessing to stabilize the feature propagation. Further, the urban function module immunity score is used as the target variable in the supervised learning task to guide the optimization process of the model. Finally, the loss function is used to measure the deviation between the predicted value and the true value, and this is used to optimize the algorithm, correct the network weights, and dynamically adjust the model parameters to improve the convergence speed of training and prediction accuracy.

#### 3.2.3 Multi-source data collection and processing

First, a data framework encompassing the dimensions of healthcare, economy, environment, and social governance is established, and the sources and methods of obtaining each type of data are clarified. For example, healthcare data include hospital capacity, healthcare worker density, and vaccination rates, while economic data encompass community collaboration levels and economic growth rates. Data sources include hospital management systems, government statistical departments, environmental monitoring platforms, public health databases, policy documents, and community management platforms. To ensure the timeliness and applicability of the data, special consideration must be given to both the data timeframe and the frequency of updates. The data timeframe spans from 2018 to 2023 and includes several PHEs (e.g., the 2019 influenza outbreak and the 2020 COVID-19 epidemic). The data are typically updated monthly; however, during major PHEs, relevant data (e.g., outbreak transmission and healthcare resource utilization) are updated more frequently, even on an hourly basis, based on real-time surveillance results. Second, a systematic data preprocessing process is implemented to ensure data quality, feature validity, and applicability for subsequent modeling. The specific steps are as follows:

(1) Data Cleaning and Anomaly Detection: The Z-score statistical method and Isolation Forest algorithm are employed for outlier identification, and extreme values (e.g., abnormal case growth, abnormal resource flow) are either eliminated or corrected. (2) Missing Value Processing: For missing data, imputation is performed using K-Nearest Neighbors Imputation (KNN), with *K* = 5, meaning that each interpolation is based on the five nearest neighbor observations. (3) Data Normalization: To eliminate differences in the scale of various indicators, Min-Max Normalization is applied to linearly compress all numerical features into the [0,1] interval, unifying the feature scale. This normalization improves the convergence speed and prediction accuracy of the deep learning model. (4) Time Series Denoising: For time series data, such as case growth and medical resource utilization, Wavelet Transform is utilized for denoising and trend extraction. (5) Feature Extraction and Dimensionality Reduction: For high-dimensional data, Principal Component Analysis (PCA) is used for dimensionality reduction, retaining the principal components that account for more than 95% of the cumulative variance. This ensures that the original information is preserved to the greatest extent while reducing dimensionality, avoiding redundancy and covariance, and enhancing the efficiency and stability of model training. (6) Multi-Source Data Fusion and Storage Management: Knowledge graph technology is employed to fuse medical, socio-economic, and policy data, constructing an urban immunity graph centered on nodes (e.g., hospitals, communities) and relationships (e.g., resource flows, policy transmission). This graph is then transformed into model inputs through KGE. A graph database and distributed storage system (Hadoop) are utilized to manage heterogeneous data from multiple sources, supporting efficient querying and subsequent GCN modeling tasks. (7) Data Annotation: Manual or semi-automatic labeling is performed for data on policy responses, resource allocation, and other factors to train supervised learning models.

#### 3.2.4 Data input and model training

First, after completing data collection and preprocessing, the structured data are input into the GNN model, and the adjacency matrix is constructed by using the adjacency relationship between urban functional modules. Second, the city immunity data are converted into a format suitable for graph neural networks, and the node feature matrix is constructed so that the model understands the city immunity features. Further, the training set and test set are defined, and the GCN is trained through supervised learning so that it can recognize the key features that affect the immunity results, and the model performance is verified on the test set. Then, the cross-validation technique is used during the training process to prevent overfitting while enhancing the predictive ability of the model through hyper-parameter optimization (e.g., adjusting the learning rate, the number of hidden layers, and the activation function). Finally, through multiple rounds of training and iterative optimization, the GCN is able to fully learn the structural features of urban immunity and is able to generate high-quality urban immunity scoring results.

#### 3.2.5 Presentation of spatial and temporal dimensions of assessment results

GIS and its related data visualization techniques are used to present the results of urban immunity assessment in an intuitive, multi-dimensional and multi-scenario manner. On the one hand, based on geospatial data, a spatial–temporal dynamic map is constructed on the urban scale, showing the differences in comprehensive immunity levels among cities and city groups, the differences in local immunity in phases and modules, and the differences in immunity-specific indexes (e.g., healthcare resources, the number of risk monitoring points, etc.). On the other hand, combined with the results of time-series data analysis, a dynamic trend map is drawn to show the changes in urban immunity at different time stages of PHEs, as well as the impact of major policies, measures and other factors on urban immunity.

### 3.3 Step 3: urban immunity optimization

Based on the aforementioned theoretical foundation and assessment results, (1) establish UIOM based on deep learning prediction and reinforcement learning optimization, (2) design simulation scenarios and output interventions, and (3) provide policy recommendations for stakeholders based on the interventions.

#### 3.3.1 Overall model architecture design

**Figure 5:**
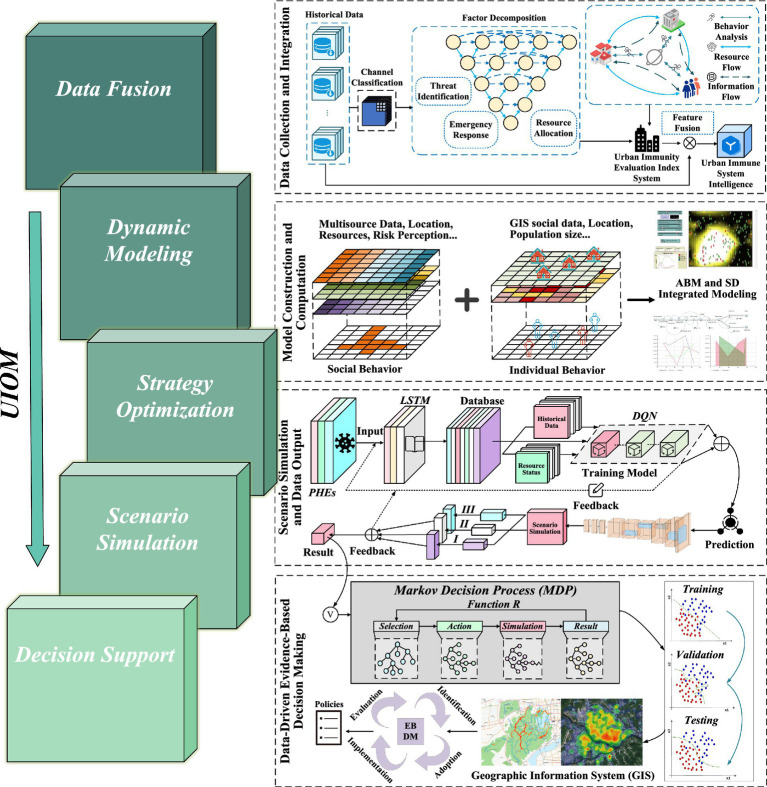
Urban immunity optimization model (UIOM).

The model consists of five core modules, including a data fusion layer, a dynamic modeling layer, a strategy optimization layer, a scenario simulation layer, and a decision support layer, as shown in Figure 5. (1) The data fusion layer is responsible for integrating the historical data of PHEs and embedding the urban immunity assessment index system, covering different dimensions such as threat identification, emergency response, and resource deployment, and establishing the urban immunity system intelligences to simulate the behavior, information flow, and resource flow of subjects such as the government, hospitals, community, and residents. (2) The dynamic modeling layer is based on the evolution process of urban immunity SD and the behavior of ABM subjects, and incorporates LSTM deep learning model to predict the future immunity strength and epidemic development trend. (3) The strategy optimization layer defines the regulation path of urban immunity through states, actions and reward mechanisms under the framework of Markov Decision Process (MDP), and optimizes the interventions of the urban immune system, such as resource allocation and policy adjustment, using DQN reinforcement learning algorithms, so that the cities to dynamically adjust their coping strategies under different scenarios. (4) The scenario simulation layer simulates different scenarios in the virtual environment to test the adaptability and effectiveness of different strategies and iteratively adjust the optimization scheme. Eventually, (5) the decision support layer presents the optimization results based on GIS to present the most intuitive urban immunity data for policy makers.

#### 3.3.2 Deep learning-based prediction module design

This module uses deep learning models to predict future immunity strength and epidemic trends. Firstly, preprocessing and constructing training sets for multi-source data, and using LSTM for time series modeling. Second, the historical PHEs data are input to output the future urban immunity prediction results. Further, the prediction results are compared with the expected target value, and if it is lower than the threshold value, the model will automatically trigger the immunity enhancement mechanism to enter the strategy optimization layer, which adjusts the prevention and control measures, such as optimizing the resource deployment and increasing the vaccination efforts, by intensively learning the time series information such as the historical epidemic data, the use of healthcare resources, and the social mobility. Finally, the new strategy is evaluated in the scenario simulation layer to predict the inflection point of epidemic development, medical resource demand, and the impact of different prevention and control measures on urban immunity.

#### 3.3.3 Reinforcement learning based strategy selection module design

Based on the LSTM prediction results, this module searches for the optimal immunity enhancement scheme through reinforcement learning under the MDP framework. First, state sets are defined, which describe the variables of the current state of the urban immune system, such as the availability of medical resources, the spread of epidemics, and social mobility. Second, design the action set, set the interventions that can be taken, such as medical resource reallocation, vaccination promotion, blockade policy adjustment, etc. Further, determine the transfer probability, which is used to characterize how the state of the urban evolves after executing a certain action, and is usually estimated based on data-driven infectious disease modeling, urban mobility analysis, or historical experience. Then, set the reward function, which is used to measure the effect of different interventions, such as reducing infection rates, reducing healthcare resource overload, and safeguarding economic stability. Next, introduce discount factors, which is used to weigh the short-term and long-term benefits. Finally, with the DQN reinforcement learning algorithm, different strategies are tried in a simulated environment and interventions are continuously optimized to maximize long-term cumulative rewards.

#### 3.3.4 Scenario simulation and data output

This module simulates and calculates and verifies the optimization scenarios by constructing different contingency scenarios to ensure the adaptability and feasibility of the strategies. First, three types of scenarios are constructed, (1) long-term optimization strategies, i.e., no major emergencies, simulating long-term immunity building, such as healthcare infrastructure optimization, public health interventions, resource reserve management, etc.; (2) emergency response strategies, simulating major epidemics or natural disaster outbreaks, testing the effectiveness of different intervention strategies, such as vaccination promotion, blockade policy adjustments, and optimization of healthcare resource allocation, etc.; (3) Damage recovery strategies, for cities to recover after an emergency, including financial subsidies, mental health interventions, and healthcare system reconstruction. Then, the model parameters are changed according to different scenarios, such as risk propagation speed, crowd mobility, policy intervention intensity, and resource allocation methods. Finally, the output results are presented on a GIS platform, while sensitivity analysis is performed on the decision-making parameters and the set of interventions is generated.

#### 3.3.5 Data-driven policy making

This section adopts the Evidence-Based Policy Making (EBP) methodology to develop a scientific, objective, and pragmatic urban immunity enhancement policy, following the process of problem identification, data analysis, policy experimentation, policy evaluation, implementation optimization, and long-term impact assessment. Initially, the vulnerabilities of cities in PHEs are analyzed from a realistic perspective, addressing issues such as insufficient healthcare resource allocation, delayed policy response, and weak community mobilization capacity. Subsequently, based on the results of scenario simulations, the effectiveness, cost-effectiveness, and socio-economic impacts of various policy options will be comprehensively assessed. During this assessment, the potential impact of administrative barriers, social resistance, and economic constraints on policy effectiveness will be considered. Finally, specific policy recommendations for public health governance will be made, taking into account feedback from multiple stakeholders, including government entities, healthcare organizations, and communities. However, the implementation of these policies may face administrative coordination challenges, particularly communication issues and resource deployment difficulties between different levels of government and departments, which could hinder the efficiency of emergency responses. To address this, resource allocation can be streamlined and response efficiency improved by establishing cross-departmental emergency working groups and adopting centralized decision-making platforms. Additionally, social acceptance remains a critical challenge during policy implementation, particularly when mandatory measures (e.g., quarantine, vaccination) encounter public resistance. To overcome this issue, it is essential to enhance policy transparency, upgrade public education efforts, and promote community participation, thereby gaining broad public support. Regarding economic resources, particularly in cases of insufficient funding, the effectiveness of policy implementation may be compromised. In such cases, funding pressures can be alleviated by collaborating with the private sector, seeking international assistance, and providing financial subsidies to ensure smooth policy execution.

## 4 Anticipated results

### 4.1 Result 1: theoretical framework construction and immune response mechanisms

This study will propose and refine the theoretical connotation and extension of urban immunity, clearly define its key elements and operational mechanisms, and form a dynamic theoretical framework that includes threat identification, alarm dissemination, emergency response, monitoring and repair, and memory feedback, so as to provide a clear and operable conceptual model for urban governance. At the same time, a two-level immune response mechanism of “inherent immunity-adaptive immunity” will be constructed to clarify the division of labor and collaboration among urban governance bodies. In addition, a standardized and replicable analytical methodology and operational process guide will be formed to guide the construction and practical application of the immune mechanism in different types of cities.

### 4.2 Result 2: assessment and presentation of results in spatial and temporal dimensions

This study will systematically construct a set of quantitative urban immunity assessment index system based on knowledge map, covering medical, economic, social and environmental fields, which includes key indicators such as efficiency of medical resources allocation, timeliness of epidemic monitoring and early warning, speed of government decision-making response and degree of community participation. At the same time, it will explicitly analyze the characteristics of the spatial and temporal distribution of urban immunity and its dynamic trend in different scenarios, and form intuitive spatial difference visualization results such as “spatial hotspot map of urban immunity” and “map of risk-sensitive areas” by GIS technology. In addition, a replicable standardized process manual for urban immunity assessment will be output, providing methodological reference and practical guidance for immunity assessment in other regions or cities.

### 4.3 Result 3: optimization of decision modeling and multi-scenario decision output

This study will establish a UIOM, integrating LSTM network prediction and DQN dynamic intervention strategy optimization methods, to explicitly output the specific effects and cost-effectiveness of interventions, such as vaccination and medical resource deployment. At the same time, this study will provide data-driven optimization scenarios for the three governance scenarios of long-term construction, short-term emergency response, and damage recovery, including specific action recommendations such as the layout of medical facilities, early warning of outbreaks, and community mobilization mechanisms. In addition, a detailed cost–benefit analysis report of the interventions will be generated, and standardized decision support tools and operational processes will be provided to assist the public health sector in effective policy formulation and precise interventions.

## 5 Discussion

Against the background of the frequent occurrence of PHEs and the inadequacy of the traditional governance model in terms of dynamic response and intelligent decision-making, this paper innovatively proposes the conceptual framework of “urban immunity,” which is modeled on the principle of the human immune system, with the aim of enhancing the resilience and adaptability of the urban governance system in risky environments. Specifically, this study designs a three-step technical path of “assessment-prediction-optimization”: first, a quantitative assessment system of urban immunity is constructed through a data-driven approach to identify the vulnerable links of the current urban immune system; second, a deep learning model is used to predict the dynamic trend of immunity change and the effect of risk propagation; finally, intelligent decision-making optimization models are constructed based on reinforcement learning to realize dynamic resource deployment and policy intervention. This study integrates social equity and ethical considerations into the development of decision-making frameworks for enhancing urban immunity. The construction of the urban immunity assessment indicator system not only considers the city’s resource allocation and policy response but also places significant emphasis on achieving equity among diverse groups, particularly in ensuring that disadvantaged groups receive additional attention when resources are limited. In implementing specific policies, it is essential to account for the unique needs and risks of different groups, with policy priorities adjusted according to the principle of social equity to ensure timely and effective protection of vulnerable populations. Furthermore, when employing a data-driven approach to policymaking, the fairness and social impact of various policy options will be regularly evaluated, and necessary adjustments will be made based on the results. This approach ensures that the study not only provides scientifically grounded, data-driven policymaking support but also fosters the social acceptability and sustainability of the policies during implementation.

This study theoretically promotes interdisciplinary innovation in urban governance and public health emergency management, proposes a new paradigm of urban public health governance that is more systematic and dynamic, and makes up for the deficiencies of traditional governance theories in whole-life-cycle management, multi-body synergy, and feedback mechanisms. In practice, it has significant theoretical and practical value to provide cities with scientific basis and technical support for accurate identification of immunization shortcomings, optimization of public resource allocation and intelligent decision support, and to promote the transformation of urban public health governance to digitalization and intelligence.

This study has several limitations. First, challenges in data acquisition and data quality may affect the accuracy and reliability of the assessment and prediction models. Second, potential variations in model applicability across different cities and national governance systems must be further verified through regional simulations and cross-city empirical studies. Specifically, the impact of differences in data standards, policy-making patterns, and psychosocial acceptance on model outputs should be considered. Additionally, the interpretability of deep and reinforcement learning models still needs to be further strengthened, which may affect the trust of decision makers and the public in the results. Finally, the urban governance system is subject to complex constraints, including data privacy protection policies, coordination barriers between governance levels, varying public acceptance of government interventions, and resource allocation conflicts, all of which must be fully considered in policy formulation and model implementation.

Future research can further strengthen cross-regional or cross-country empirical comparisons to validate the universality of theories and methods; at the same time, improve the transparency and credibility of the models by introducing interpretable artificial intelligence techniques; and explore the combination with digital twin technology to develop real-time decision support systems. In addition, the multidisciplinary integration of urban immunity theory with other urban governance theories should be promoted, and the comprehensive assessment of socio-economic and ethical impact dimensions should be added, so as to realize a more comprehensive, precise and socially acceptable public health governance.

## Data Availability

The original contributions presented in the study are included in the article/supplementary material, further inquiries can be directed to the corresponding author.
